# Third Chromosome Balancer Inversions Disrupt Protein-Coding Genes and Influence Distal Recombination Events in *Drosophila melanogaster*

**DOI:** 10.1534/g3.116.029330

**Published:** 2016-06-28

**Authors:** Danny E. Miller, Kevin R. Cook, Alexandra V. Arvanitakis, R. Scott Hawley

**Affiliations:** * Stowers Institute for Medical Research, Kansas City, Missouri 64110; † Department of Molecular and Integrative Physiology, University of Kansas Medical Center, Kansas City, Kansas 66160; ‡ Department of Biology, Indiana University, Bloomington, Indiana 47405

**Keywords:** balancer chromosomes, p53, meiosis, whole-genome sequencing, crossing over

## Abstract

Balancer chromosomes are multiply inverted chromosomes that suppress meiotic crossing over and prevent the recovery of crossover products. Balancers are commonly used in *Drosophila melanogaster* to maintain deleterious alleles and in stock construction. They exist for all three major chromosomes, yet the molecular location of the breakpoints and the exact nature of many of the mutations carried by the second and third chromosome balancers has not been available. Here, we precisely locate eight of 10 of the breakpoints on the third chromosome balancer *TM3*, six of eight on *TM6*, and nine of 11 breakpoints on *TM6B*. We find that one of the inversion breakpoints on *TM3* bisects the highly conserved tumor suppressor gene *p53*—a finding that may have important consequences for a wide range of studies in *Drosophila*. We also identify evidence of single and double crossovers between several *TM3* and *TM6B* balancers and their normal-sequence homologs that have created genetic diversity among these chromosomes. Overall, this work demonstrates the practical importance of precisely identifying the position of inversion breakpoints of balancer chromosomes and characterizing the mutant alleles carried by them.

Balancer chromosomes are multiply rearranged chromosomes that are used extensively in *Drosophila melanogaster* for tasks such as stock construction and the maintenance of recessive deleterious alleles in populations (Ashburner *et al.* 2005). Balancers work by suppressing meiotic crossing over, by creating recombinant chromatids that will not segregate properly during the first meiotic division (Novitski and Braver 1954), or, in the case of pericentric inversions, by creating recombinants that carry duplications or deficiencies large enough to result in zygotic lethality. While all balancer chromosomes carry easily scored dominant marker alleles that allow for visual identification of flies carrying the balancer, most balancers also carry recessive lethal mutations that prevent the balancer from becoming homozygous in stock (Lindsley and Zimm 1992; Ashburner *et al.* 2005).

 A variety of balancers are available for the X, second, and third chromosomes in *Drosophila*, and they have become increasingly effective as the number of inversions has increased and as visible markers and recessive lethal or sterile alleles have been added. For example, *First Multiple one* (*FM1*), an X chromosome balancer, improved upon earlier single-inversion balancers such as *In(1)dl-49*, *In(1)sc*, and *ClB*, by combining the *In(1)dl-49* and *In(1)sc* inversions into one chromosome (Lindsley and Zimm 1992; Ashburner *et al.* 2005). Further improvements generated a series of FM balancers, and similar series exist for the second (second multiple; SM) and third (third multiple; TM) chromosomes (Lindsley and Zimm 1992). The current study will focus on the third chromosome balancers *TM3*, *TM6*, and *TM6B*.

*TM3* was created in the late 1950s by repeated X-raying of a chromosome marked with *kni^ri-1^*, *p^p^*, *vvl^sep^* (previously known as *sep^1^*), *Ubx^bx-34e^*, and *e^1^*, and carrying two inversions, *In(3LR)sep* (65D2-3;85F2-4) and *In(3R)C* (92D1-E1;100F2-3). The irradiation superimposed three additional inversions on this chromosome, creating a balancer with five total inversions (Lewis 1960) (Figure 1). Tinderholt (1960) introduced the dominant markers *Serrate* (*Ser*) and *Stubble* (*Sb*) into inverted regions of this chromosome by double crossing over, relying on the increased recombination created by the so-called interchromosomal effect (Schultz and Redfield 1951; Ramel 1966) to obtain these double crossovers (DCOs). Specifically, Tinderholt (1960) performed this synthesis in a female heterozygous for three balancers to increase the likelihood of recombination within the desired inversions. In doing so, he demonstrated that segments could be swapped into inverted segments of *TM3*—even if such events were uncommon.

**Figure 1:**
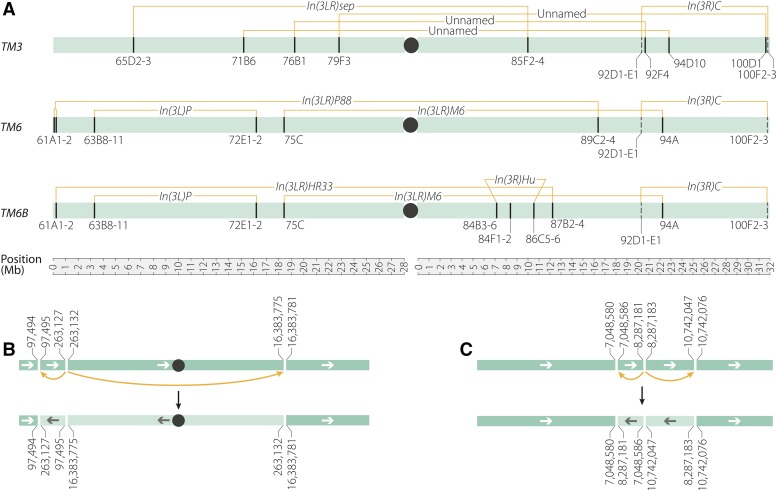
*TM3*, *TM6*, and *TM6B* inversion breakpoints. Black circles indicate centromeres and left-facing arrows indicate an inverted segment. (A) The inversions carried by the third chromosome balancers *TM3*, *TM6*, and *TM6B*. Breakpoints that have been molecularly identified are shown as solid lines; those that are estimates are shown as dashed lines; numbers are cytological bands of breakpoints given in Lindsley and Zimm (1992). (B) The *In(3LR)P88* (61A­1-2;89C2-4) rearrangement on *TM6* is a previously unreported three-breakpoint rearrangement with a breakpoint at *3L*:263,127–263,132 that bisects the gene *Tudor-SN*, a breakpoint at *3R*:16,383,781 that bisects *spineless*, an allele previously reported to be carried by this chromosome (Duncan *et al.* 1998), and a breakpoint at *3L*:97,494 that is intergenic. (C) In the *In(3R)Hu* (84B1;84F4;86C7-8) three-breakpoint rearrangement on *TM6B*, the breakpoint at *3R*:8,287,181 bisects the noncoding RNA gene *CR44318* while the *3R*:10,742,076 breakpoint bisects *TkR86C*. The breakpoint at 3R:7,048,580 likely causes the *Antp^Hu^* phenotype.

The progenitor chromosome that was X-rayed to produce *TM3* also carried *Dp(1;3)sc^260-20^*, an aberration that replaced the tip of chromosome *3L* with the tip of an X chromosome carrying a wild-type allele of the *yellow* (*y*) gene (Sutton 1943). However, this *y^+^* marker was frequently lost by a single crossover event between *TM3* and normal-sequence chromosomes in the region distal to the 65D inversion breakpoint; consequently, most *TM3* chromosomes now carry a normal *3L* tip (Shearn 1980). This is one of several observations indicating that the relatively large uninverted region distal to 65D undergoes frequent exchange events—even though recombination is largely suppressed in regions proximal to 65D.

*TM6* was created by X-ray mutagenesis of a chromosome marked with *Ubx^bx-34e^* and *e^1^* and carrying three preexisting inversions: *In(3L)P* (63C;72E1-2) lying inside *In(3LR)P88* (61A;89CD) with *In(3R)C* (92D1-E1;100F2-3) to the right (Figure 1). Irradiation resulted in an additional inversion, *In(3LR)M6*, between bands 75C and 94A (Lindsley and Zimm 1992). *TM6B* was built from *TM6* by replacing the left breakpoint of *In(3LR)P88* with the left end of *In(3LR)HR33* (61A1-2;87B) (Ashburner 1972) by a single crossover (Figure 1). The three-breakpoint rearrangement *In(3R)Hu* (84B1;84F4;86C7-8) (Hazelrigg and Kaufman 1983) was carried onto the recombinant chromosome along with the left end of *In(3LR)HR33* from a double-aberration progenitor. An internal segment spanning the right breakpoint of *In(3LR)P88* was then replaced with a segment spanning the right breakpoint of *In(3LR)HR33* by a DCO. Finally, the dominant *Tubby* (*Tb^1^*) marker was added by a DCO event within an inverted segment near the right end of the newly created *TM6B* (Craymer 1981, 1984; Lindsley and Zimm 1992).

Because balancers are used widely in *Drosophila* experiments, sometimes as heterozygous controls, it is informative for the community to determine the exact position of their breakpoints and the nature of the alleles carried by them. A recent study reported rare DCO events between the X chromosome balancer *FM7* and its normal sequence homologs that were selected for because they conferred an advantage to flies carrying the recombinant chromosome (Miller *et al.* 2016a). A similar whole-genome analysis of commonly used autosomal balancers has not yet been conducted.

Here, we use whole-genome sequencing to identify all but one shared pair of inversion breakpoints on the *TM3*, *TM6*, and *TM6B* balancer chromosomes (Figure 1). Importantly, we find that the breakpoint at 94D on *TM3* splits the highly conserved tumor suppressor gene *p53* in half, demonstrating that any stock balanced with *TM3* is heterozygous for a *p53* loss-of-function allele. We also find evidence of single and double crossover events on more than half of the *TM3* chromosomes sampled and on one *TM6B* chromosome, and we are able to estimate the distance over which inversions suppress exchange by examining single crossover events that occur in an unbalanced region of the *TM3* chromosome. These findings demonstrate that, similar to the *X* chromosome balancer *FM7*, sequence diversity exists among third chromosome balancers and suggest that this variation may influence experimental outcomes.

## Materials and Methods

### Stocks used for breakpoint identification and validation

Stocks used in this study, along with their current Bloomington IDs and genotypes, are listed in Supplemental Material, Table S2. Throughout the manuscript we have referred to stocks as <balancer chromosome>-<Bloomington Stock ID> unless they were lab stocks not available at the Bloomington stock center, in which case they are referred to as <balancer chromosome>-lab. For example, *TM3-500* is the *TM3* chromosome in Bloomington stock 500. The ISO-1 (*y^1^*; *Gr22b^iso-1^ Gr22d^iso-1^ cn^1^ CG33964^iso-1^ bw^1^ sp^1^*; *LysC^iso-1^ MstProx^iso-1^ GstD5^iso-1^ Rh6^1^*) stock used to create heterozygous *TM3* and *TM6B* flies for sequencing was obtained from Sue Celniker (Lawrence Berkeley National Laboratory). The single *TM6* chromosome used in this study was not sequenced as an ISO-1/*TM6* heterozygote, but as a +/*TM6* heterozygote. All flies were kept on standard cornmeal-molasses medium and maintained at 25°.

### DNA preparation and genome alignment

DNA for sequencing was prepared from either heterozygous males or a combination of heterozygous males and heterozygous females using the Qiagen DNeasy Blood and Tissue Kit. All flies were starved for 1 hr before freezing at –80° for at least 1 hr. Mate pair DNA libraries for stocks *CyO-TM3-mp-22239*, *SM6a-TM3-mp-lab*, and *TM6B-mp-587* were generated from 1 μg of high-quality genomic DNA. Following the manufacturer’s directions, libraries were generated using the gel-free method of the Illumina Nextera Mate Pair Library Preparation kit, with 10 cycles of PCR amplification. Resulting libraries were checked for quality and quantity using a Bioanalyzer 2100 (Agilent) and Qubit Fluorometer (Life Technologies). All libraries were pooled, requantified, and sequenced as 150-bp paired end on an Illumina NextSeq 500 instrument. Following sequencing, Illumina Real Time Analysis version 2.4.6 was run to demultiplex reads and generate FASTQ files; 100 ng of sample *TM6-lab* was sheared using the Covaris s220 instrument to 300 bp, and prepared using the KAPA HTP Library Prep Kit for Illumina and Bioo Scientific NEXTflex DNA barcodes. The resulting library was quantified using a LabChip GXII (Perkin Elmer), and a Qubit Fluorometer (Life Technologies). This library was pooled with others, requantified and sequenced as 150-bp paired end on an Illumina HiSeq 2500 in rapid mode. Following sequencing, Illumina Real Time Analysis version 1.17.21.3 and CASAVA version 1.8.2 were run to demultiplex reads and generate FASTQ files. For the remainder of samples used in this study, 500 ng of DNA from each was fragmented to 600-bp fragments using a Covaris S220 sonicator by adjusting the treatment time to 30 sec, except for sample *CyO-TM3-504*, which was sonicated using 89 ng of DNA and was not size selected. Libraries were prepared using the KAPA HTP Library Prep Kit for Illumina and Bioo Scientific NEXTflex DNA barcodes. The resulting libraries were quantified using a Bioanalyzer (Agilent Technologies) and a Qubit Fluorometer (Life Technologies). All libraries were pooled, requantified, and sequenced as 150-bp paired end on the Illumina NextSeq 500 instrument. Following sequencing, Illumina Real Time Analysis version 2.4.6 was run to demultiplex reads and generate FASTQ files. Alignment to the *D. melanogaster* reference genome (dm6) was performed using bwa version 0.7.7-r441 (Li and Durbin 2009). SNPs were called using SAMtools and BCFtools (version 0.1.19-44428cd) (Li *et al.* 2009). Indels were not considered and low quality SNPs (those with quality scores < 200) were filtered out. All *TM3* and *TM6B* chromosomes were sequenced as balancer/ISO-1, allowing us to treat every heterozygous SNP as a SNP present on the balancer chromosome.

### Identification and validation of inversion breakpoints

Breakpoints were identified as reported in Miller *et al.* (2016a). Briefly, split or discordant read pairs were isolated using Samblaster (Faust and Hall 2014), and known regions of repetitive or low-complexity sequence were masked with repeatmasker (Chen 2004). Separately, we used BreakDancer (Chen *et al.* 2009) to identify candidate inversion breakpoints. Regions where BreakDancer identified large inversion polymorphisms and where rearrangements were previously reported to be present (Lindsley and Zimm 1992) were analyzed in 1-kb windows for regions that contained more than 10 split or discordant read pairs. Breakpoints were visually validated using Integrative Genomics Viewer (Thorvaldsdottir *et al.* 2013) and the UCSC Genome Browser (Rosenbloom *et al.* 2015). Original FASTQ reads from each breakpoint were collected and *de novo* assembled using SOAPdenovo2 with a kmer size of 41 (Luo *et al.* 2012). Primers for PCR validation were designed using Primer3 (Rozen and Skaletsky 2000). PCR was done with Phusion polymerase, and Sanger sequencing confirmed each breakpoint. PCR primers used to validate inversion breakpoints are listed in Table S1.

### Identification of crossover events and generation of heatmaps

**Figure 2:**
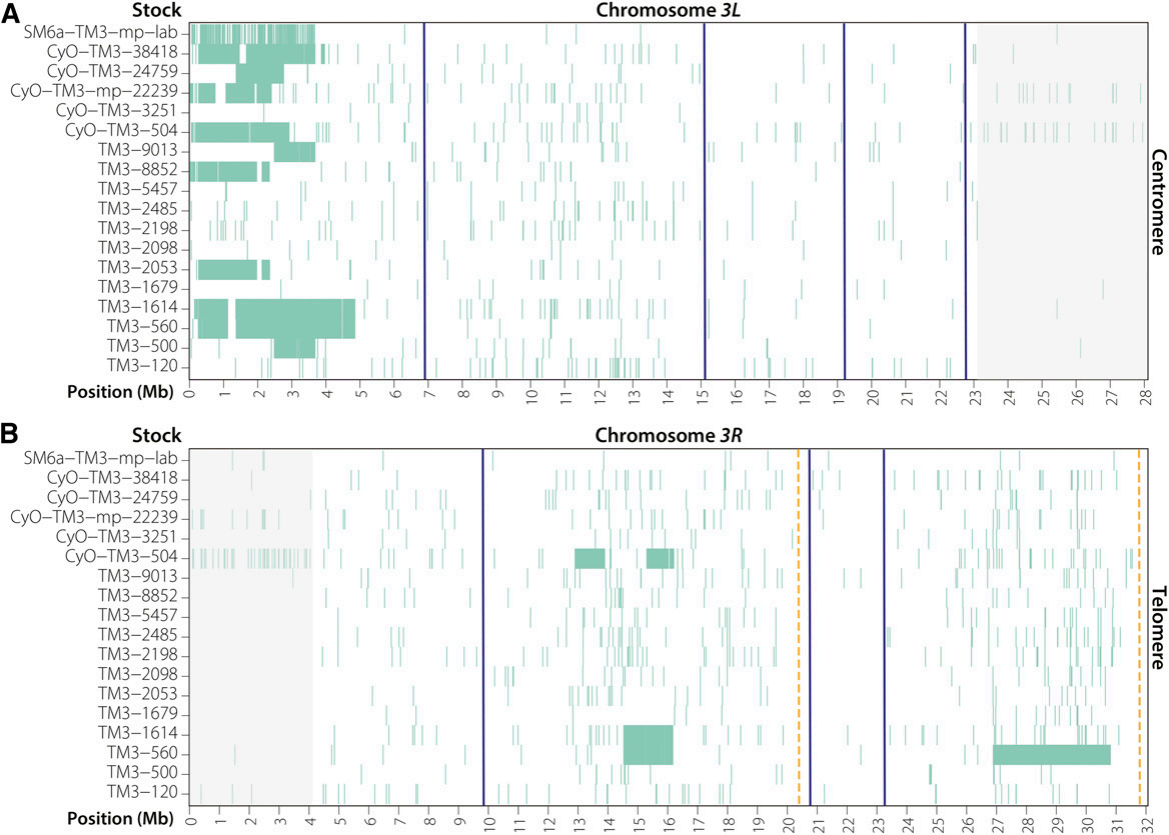
Visualizing SNPs present in five or fewer *TM3* chromosomes reveals numerous single crossover events on *3L* and several DCO events on *3R* (see *Materials and Methods*). Blue lines indicate the positions of inversion breakpoints whose precise location is known, orange dashed lines show the approximate positions of the unidentified *In(3R)C* (92D1-E1;100F2-3) inversion breakpoints. Gray shaded regions are centromere-proximal heterochromatin with low-quality read alignment. (A) Single crossovers are common in the region distal to the 65D inversion breakpoint at position *3L*:6,925,034, and occur no closer than 2 Mb from the breakpoint. (B) Several DCOs are apparent on *3R*. Stocks *TM3-560* and *TM3-1614* may be versions of *TM3* before *Ser^1^* was added to a *TM3*, *Sb^+^ Ser^+^* chromosome (*TM3-560*), and before *Sb^1^* was added to a *TM3*, *Sb^+^ Ser^1^* chromosome (*TM3-1614*).

**Figure 3:**
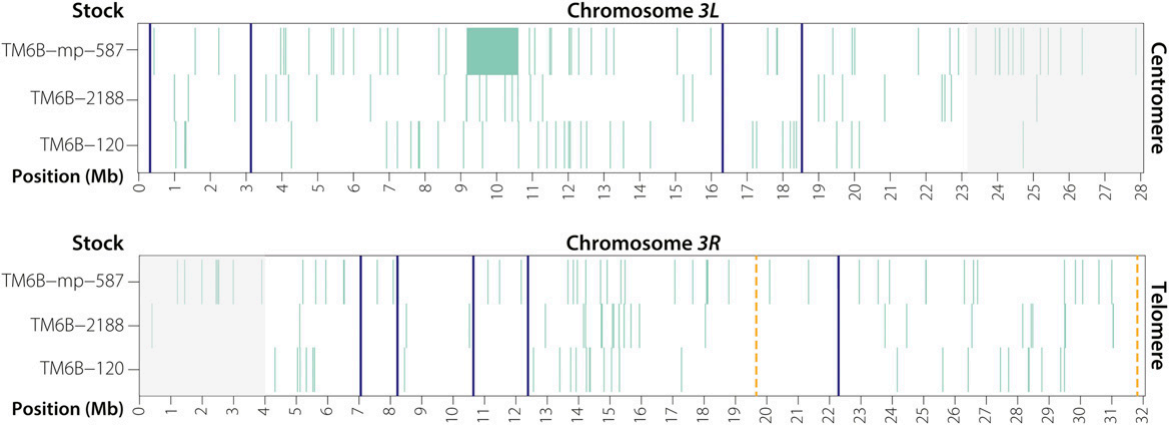
Unique SNPs present among the three *TM6B* chromosomes sequenced in this study. Gray shaded regions are centromere-proximal heterochromatin with low-quality read alignment. Blue lines indicate the positions of inversion breakpoints, orange dashed lines indicate the approximate positions of the unidentified *In(3R)C* (92D1-E1;100F2-3) inversion breakpoints. A single DCO event was recovered in stock *TM6B-mp-587*.

Because no *TM3* or *TM6B* reference sequence exists, we identified crossover tracts by comparing the SNP profile of each individual chromosome to all chromosomes of the same type. Specifically, for each SNP from each stock, we checked all other stocks to see if the same polymorphism was present. If it was, then this was considered a variant carried by all balancers of that type. If a SNP was observed in only one stock, then we considered it a unique polymorphism. To build the heatmap in Figure 2, we plotted SNPs in 10-kb windows if that SNP was present in five or fewer *TM3* stocks. The interval on *TM3* between the telomere and the 65D breakpoint at *3L*:6,925,034 contained ∼43,000 informative SNPs in each of the *TM3* stocks sequenced. For the three *TM6B* stocks represented in Figure 3, we plotted completely unique SNPs, or any SNP not shared by all three chromosomes.

### Data availability

Raw sequencing data for all samples used in this project have been uploaded to the National Center for Biotechnology Information (NCBI) at ncbi.nlm.nih.gov and can be found under BioProject PRJNA315473. Laboratory strains of *wg^Sp-1^*/*SM6a*, *Duox^Cy^*; *Pr^1^*/*TM3*, *Sb^1^ Ser^1^*, and *+*/*TM6* are available upon request; all other strains listed in Table S2 are available from the Bloomington Drosophila Stock Center.

## Results

Using whole-genome sequencing, we precisely identified eight of the 10 breakpoints on *TM3*, six of eight breakpoints on *TM6*, and nine of 11 breakpoints on *TM6B* (Figure 1A and Table 1). The three balancers share an inversion, *In(3R)C*, between cytological bands 92D1-E1 and 100F2-3 (Sturtevant 1913; Muller 1918) that we were unable to accurately position because its location near the telomere suggests that it most likely involves highly repetitive sequences. Note that, throughout the manuscript, we refer to breakpoints by the names of the inversions that created them. We also use the observed cytological bands reported in Lindsley and Zimm (1992) rather than the estimated cytological bands that are available on FlyBase or the UCSC genome browser.

**Table 1:** Molecular details of the *TM3*, *TM6*, and *TM6B* inversion breakpoints

Balancer	Inversion	Chr	Reported bands*^a^*	5′ Break	3′ Break	Duplication (+) /Deletion (-)	Affected Gene/Region
*TM3*	*In(3LR)sep*	*3L*	65D2-3	6,925,034	6,926,125	−1090	Intergenic
		*3R*	85F2-4	9,943,831	9,944,040	−208	*Glut4EF*
*TM3*	Unnamed	*3L*	71B6	15,150,269	15,150,272	−2	*FucTA*
		*3R*	94D10	23,050,763	23,050,764	0	*p53*
*TM3*	Unnamed	*3L*	76B1	19,386,273	19,388,151	−1877	*CG32206*, *ms(3)76Ba*
		*3R*	92F4	20,637,930	20,637,930	+1	*Lrrk*
*TM3*	Unnamed	*3L*	79F3	22,637,876	22,637,952	−75	*CG14459*
		*3R*	100D1	31,653,695	31,653,707	−11	*kek6*
*TM3*, *TM6*, *TM6B*	*In(3R)C*	*3R*	92D1-E1	Unknown	Unknown	—	Unknown
		*3R*	100F2-3	Unknown	Unknown	—	Unknown
*TM6*	*In(3LR)P88*	*3L*	61A1-2	97,494	97,495	0	Intergenic
		*3L*	61A1-2	263,127	263,132	−4	*Tudor-SN*
		*3R*	89C2-4	16,383,781	16,383,775	+7	*ss*
*TM6*, *TM6B*	*In(3LR)M6*	*3L*	75C	18,693,657	18,693,663	−5	*CR43987*
		*3R*	94A	22,393,827	22,393,828	0	*CG13857*
*TM6*, *TM6B*	*In(3L)P*	*3L*	63B8-11	3,173,046	3,173,053	−6	*CG14964*
		*3L*	72E1-2	16,308,841	16,308,845	−3	Intergenic
*TM6B*	*In(3LR)HR33*	*3L*	61A1-2	233,562	233,565	−2	Intergenic
		*3R*	87B2-4	12,227,473	12,227,471	+3	Intergenic
*TM6B*	*In(3R)Hu*	*3R*	86C5-6	10,742,047	10,742,076	−28	*TkR86C*
		*3R*	84F1-2	8,287,181	8,287,183	−1	*CR44318*
		*3R*	84B3-6	7,048,580	7,048,586	−5	Intergenic

a Reported bands are those found in Lindsley and Zimm (1992), and are not based on estimated genomic position.

Because autosomal balancers carry recessive lethal mutations, the recovery of homozygous progeny for sequencing is not feasible. To circumvent this problem, we crossed males from each *TM3* and *TM6B* balancer stock to females from the ISO-1 stock that was used to construct the *Drosophila* reference genome and recovered heterozygous individuals for sequencing (see *Materials and Methods*). We confirmed breakpoints by two methods: first, we whole-genome sequenced large-insert (2–12 kb) library preparations for two *TM3* stocks and one *TM6B* stock (see *Materials and Methods*); second, we PCR- and Sanger-sequenced all identified breakpoints on *TM3* and *TM6*, and selected breakpoints on *TM6B* (Table S1).

### Third chromosome balancer breakpoints disrupt protein-coding genes

After identifying the exact position of each inversion breakpoint, we found that the breakpoints on *TM3* altered six characterized [*Glut4EF*, *FucTA*, *p53*, *ms(3)76Ba*, *Lrrk*, and *kek6*], and two uncharacterized (*CG32206* and *CG14459*) protein-coding genes (Table 1). Perhaps most importantly, we observed that the 94D inversion breakpoint on *TM3* at *3R*:23,050,763–23,050,764 bisects the fifth intron of the highly conserved tumor suppressor *p53* (Jin *et al.* 2000) and affects all reported *p53* isoforms. We also confirmed that the allele *Glut4EF^TM3^* is caused by the inversion at 85F2 on *TM3*, as reported by Yazdani *et al.* (2008) (Table 2). Finally, we found that the *y^+^* X chromosome fragment originally present on *TM3* (Lewis 1960; Shearn 1980) was the result of a break of the X chromosome at *X*:416,997 and subsequent attachment to the third chromosome at *3L*:149,709, in agreement with its original isolation as a reciprocal translocation affecting the X-linked *scute* gene (Sutton 1943). This rearrangement deletes or disrupts 10 protein-coding and eight noncoding RNA genes from the third chromosome in the distal 150-kb interval of *TM3*.

**Table 2.** Genomic aberrations of previously characterized mutations and recessive lethal alleles carried by *TM3, TM6*, and *TM6B*

Gene	Allele	Balancer(s)	Observed aberration	Previous reports
*ebony*	*e*^*1*^	*TM3*, *TM6*, *TM6B*	TE (family: 412) at *3R*:21,231,832–21,231,838, 6 nt into the 2nd exon	—
*Ultrabithorax*	*Ubx*^*bx-34e*^	*TM3*, *TM6*	TE (family: DMIS176) insertion in the first intron of *Ubx* at approximately *3R*:16,731,980	Gypsy insertion (Bender *et al.* 1983)
*knirps*	*kni*^*ri-1*^	*TM3*	252-bp deletion at *3L*:20,707,101-20,707,352.	(Lunde 2003)
*pink*	*p*^*p*^	*TM3*	1-bp deletion at *3R*:6,661,619 resulting in a frameshift	1-bp deletion at *3R*:6,661,624 (Syrzycka *et al.* 2007)
*lethal (3) 89Aa*	*l(3)89Aa*^*1*^	*TM3*	Unknown	Mapped to 89A2-89A5
*ventral veins lacking*	*vvl*^*sep*^	*TM3*	Unknown	—
*Stubble*	*Sb*^*1*^	*TM3*	TE (family: 412) insertion in 4th exon of *Sb* at *3R*:16,141,939-16,141,942.	TE insertion (Hammonds and Fristrom 2006)
*Serrate*	*Ser*^*1*^	*TM3*	TE (family: TIRANT) insertion at *3R*:27,172,910-27,172,913 in the 3’ UTR of *Ser*	TE insertion (Fleming *et al.* 1990)
*Ultrabithorax*	*Ubx*^*P15*^	*TM6*	Unknown	—
*Henna*	*Hn*^*P*^	*TM6*	Multiple deletions within the first intron and a G->A mutation at splice acceptor site (AG becomes AA) in the third intron of the gene.	—
*spineless*	*ss*^*aP88*^	*TM6*	Gene is split by the *In(3LR)P88* (61A1-2;89C2-4) rearrangement.	Break in the transcription unit (Duncan *et al.* 1998)
*Antennapedia*	*Antp*^*Hu*^	*TM6B*	Unknown. Phenotype may be a result of the *In(3R)Hu* triple rearrangement (Figure 1).	—
*Tubby*	*Tb*^*1*^	*TM6B*	An in-frame 15-nt deletion in the 2^nd^ exon from *3R*:26,656,728-26,656,742; a 69-nt in-frame deletion of 23 amino acids from *3R*:26,657,089-26,657,157; and a T->G mutation (Ser->Ala) at *3R*:26,657,334.	—

 The breakpoints on *TM6* affected four protein-coding genes (*Tudor-SN*, *ss*, *CG13857*, and *CG14964*) and one noncoding RNA gene (*CR43987*) (Table 1). Using whole-genome data, we confirmed that the previously reported *spineless* allele (*ss^aP88^*) on *TM6*, reported as a break in the transcription unit (Duncan *et al.* 1998), is indeed caused by the inversion at 89C4 (Table 2). We also observed that the *In(3LR)P88* (61A;89CD) inversion on *TM6*, which had been reported to be a simple inversion of 61A to 89C, is instead a three-breakpoint rearrangement that creates a previously unknown 165-kb inversion (Figure 1B and Table 1).

Finally, the *TM6B* breakpoints affect three protein-coding genes (*CG13857*, *CG14964*, and *TkR86c*) and two noncoding RNA genes (*CR43987* and *CR44318*) (Table 1). We also characterized the three-breakpoint *In(3R)Hu* (84B1;84F4;86C7-8) rearrangement on *TM6B*, and found that it consists of 1.2-Mb and 2.5-Mb inverted segments (Figure 1C and Table 1). Based on the positions of these breakpoints, it appears that the gain-of-function mutation *Antennapedia^Hu^* (*Antp^Hu^*) is a regulatory mutation caused by the 84B1 inversion breakpoint that lies ∼50 kb away from *Antp*, as suggested by previous breakpoint mapping (Hazelrigg and Kaufman 1983; Scott *et al.* 1983).

In addition to the mutations caused by inversion breakpoints, balancer chromosomes carry a number of mutations that provide visible markers for easy identification, as well as recessive lethal alleles that prevent balancers from becoming homozygous in stock. Some of these markers are shared by more than one balancer—such as *ebony* (*e^1^*), present on *TM3*, *TM6*, and *TM6B*—while others are present on only one balancer—such as *Tubby* (*Tb^1^*), present only on *TM6B* (Table 2). The general nature of many of these alleles has been described previously [such as that a transposable element (TE) insertion in *Ultrabithorax* gives rise to the *Ubx^bx-34e^* allele carried by *TM3* and *TM6* (Bender *et al.* 1983), or that a TE insertion is responsible for *Ser^1^* on *TM3* (Fleming *et al.* 1990)], but the specific lesions that convey their respective phenotypes are unknown for most alleles. Using our whole-genome sequencing data, we were able to identify the precise nature of nine of 13 visible or lethal alleles carried by the three balancers analyzed in this study. These data are summarized in Table 2.

### The TM3 balancer allows single crossover events distal to 65D

Inversion breakpoints are known to suppress exchange in nearby regions, but the mechanism by which they do this, and over what distance they act, is unknown (Sturtevant and Beadle 1936; Novitski and Braver 1954). Previous work has shown that balancer chromosomes pair along their lengths with their normal-sequence homologs (Gong *et al.* 2005) and that both crossover-associated and noncrossover gene conversion events occur between balancers and their normal-sequence homologs (Blumenstiel *et al.* 2009; Miller *et al.* 2016a). Because the distal-most inversion breakpoint on the left arm of *TM3* is 6.9 Mb from the telomere (estimated cytological band 65D3), we hypothesized that single crossover events would be common in this region (Figure 1A). Evidence of recombination within this interval would manifest as tracts of unique SNPs within a pool of *TM3* chromosomes; thus, we sequenced a panel of 17 stocks from the Bloomington Drosophila Stock Center and one laboratory stock carrying the *TM3* chromosome (Table S2) to identify how close to the inversion breakpoint these crossovers occurred.

We saw evidence of crossing over between the telomere and the most distal *3L* inversion breakpoint in 11 of 18 *TM3* stocks (Figure 2). Because the ancestral SNP profile of the *TM3* chromosome is unknown, we can infer recombination events by identifying SNPs that are unique to only one of the *TM3* chromosomes. However, because of the potential relatedness of some chromosomes, we plotted SNPs that were shared by five or fewer *TM3* chromosomes. Several stocks in Figure 2A appear to have large gaps lacking SNPs in the intervals of recombination. This is likely evidence of multiple single exchange events in which a *TM3* and a normal-sequence third chromosome exchanged distal regions, suggesting that exchange distal to 65D is an ongoing process in stocks.

Several of the crossover tracts recovered are shared among multiple stocks, highlighting the relatedness of these chromosomes. For example, stocks *TM3-560* and *TM3-1614* share identical SNPs in the 1- to 5-Mb interval of *3L*, and stocks *TM3-500* and *TM3-9013* are nearly identical over 2.5–3.5 Mb in this same region (Figure 2A). Finally, crossovers in stocks *TM3-560* and *TM3-1614* are observed as close as ∼2 Mb from the inversion breakpoint, the first evidence of the distance over which an inversion breakpoint may suppress exchange.

### Double crossover events can occur on **TM3** and **TM6B**

We were also able to identify DCOs that had occurred within inverted segments on both the *TM3* and *TM6B* balancers. We found three DCO events that replaced a mutant copy of *Stubble* (*Sb^1^*) with a wild-type copy in stocks that are phenotypically *Sb^+^* (Figure 2B). Based on shared SNPs, the 1.7-Mb segment between 14.5 Mb and 16.2 Mb in *TM3-1614* and *TM3-560* appears to have originated from a single DCO in a common progenitor, while the 900-kb segment in *CyO-TM3-500* likely arose by an independent DCO event. In addition, we also found a 3.9-Mb DCO that replaced a mutant copy of *Serrate* (*Ser^1^*) with a wild-type copy (Figure 2B). While difficult to confirm, *TM3-560* may be directly related to the original isolate of *TM3* before *Ser^1^* and *Sb^1^* were added by Tinderholt (1960), and *TM3-1614* may be the *Sb^+^ Ser^1^* version of the chromosome after *Ser^1^* was added and before *Sb^1^* was added (Tinderholt 1960). *Ser^1^* and *Sb^1^* were introduced intentionally by screening for DCOs in the presence of additional balancers, which strengthen the interchromosomal effect on recombination.

The *TM3* chromosome carried by the *CyO-TM3-504* stock carries a second 1-Mb DCO event near the DCO that replaced *Sb^1^* with *Sb^+^* (Figure 2B). Analysis of this region using SnpEFF (Cingolani *et al.* 2012) finds no obvious deleterious mutations in this interval on any other *TM3* chromosome. We do, however, find a 10-kb tandem duplication within this DCO that fully duplicates *CG31157* and *CG7966*, two uncharacterized genes highly expressed in a variety of tissues, which may confer a competitive advantage to flies carrying the duplication. Interestingly, *CG7966*, which encodes a selenium-binding protein, is conserved from *Drosophila* to humans (SELENBP1), which makes this duplication a provocative candidate for further study.

The two presumed DCO events on *CyO-TM3-504* are also interesting because of their sizes. At 900 kb and 1 Mb, these are likely the smallest DCO events yet reported in Drosophila—even smaller than the 1.5-Mb DCO observed in a recent study (Miller *et al.* 2016b). It is unlikely these two DCOs are the result of a single larger DCO at coordinates 12.9–16.2 Mb followed by a second DCO at coordinates 13.9–15.3 Mb, because the second DCO would have had to occur with a homologous *TM3* or *TM3* progenitor chromosome. A simpler explanation is that these were two independent DCOs.

Finally, we identified a single 1.4-Mb DCO at *3L*:9,216,999–10,625,261 on *TM6B-587* (Figure 3). It replaces three separate frameshifting deletions in the uncharacterized genes *CG46121*, *CG16711*, and *CG32055* with wild-type copies—a potential advantage for flies carrying this chromosome. Overall, our findings provide molecular evidence that, while rare, DCO events do occur between *TM3* or *TM6B* balancers and their normal-sequence homologs.

## Discussion

We have identified the precise locations of all inversion breakpoints from the *Drosophila* third chromosome balancers *TM3*, *TM6*, and *TM6B* except for the *In(3R)C* (92D1-E1;100F2-3) inversion shared by all three chromosomes. We find that one of the *TM3* inversion breakpoints bisects all transcripts of the tumor suppressor gene *p53*, with implications for a wide range of studies in *Drosophila*. As hypothesized, we identified evidence of single crossover events in the 6.9-Mb interval between the telomere and the most distal inversion breakpoint on *TM3* in nearly two-thirds of the stocks we sequenced. These single crossover events provide the first evidence for the distance over which inversion breakpoints can suppress meiotic exchange in *Drosophila*.

Eleven of 18 *TM3* stocks carried evidence of a recombination event between the 65D breakpoint and the telomere, with the closest exchange event occurring ∼2 Mb from the 65D breakpoint. Do all inversion breakpoints suppress exchange in a similar way and over a similar distance? Perhaps the most instructive case is that of the *X* chromosome inversion *In(1)dl-49*. The distal-most breakpoint of the inversion lies ∼4.9 Mb from the telomere (2 Mb closer to the telomere than the 65D breakpoint on *TM3*). Recombination in a single generation was previously measured between the distal-most breakpoint of *In(1)dl-49* and the telomere using *yellow*, a marker near the telomere, and *echinus*, a marker ∼1 Mb from the most distal *In(1)dl-49* breakpoint, and was found to be ∼10% of what it would be in the absence of the inversion (Stone and Thomas 1935; Sturtevant and Beadle 1936). Although we did recover a substantial number of *TM3* chromosomes that had undergone distal exchanges, it must be remembered that these could have occurred at any point in the history of each *TM3* balancer. While not examined here, it would be interesting to see if recombination is reduced between 65D and the telomere on *TM3* within a single generation; we would indeed predict such a reduction. Alternatively, future studies using methods similar to ours could determine exactly how close to other inversions, such as *In(1)dl-49*, recombination can occur. Either way, the consequence for balanced chromosomes remains the same—crossing over is possible within this region. One feasible explanation for the high diversity in the region distal to 65D observed among the panel of *TM3* chromosomes we sampled is that exchange events may confer a competitive advantage in this region and can propagate throughout a stock, although the exact advantage of a recombinant *TM3* chromosome remains unclear.

**Figure 4:**
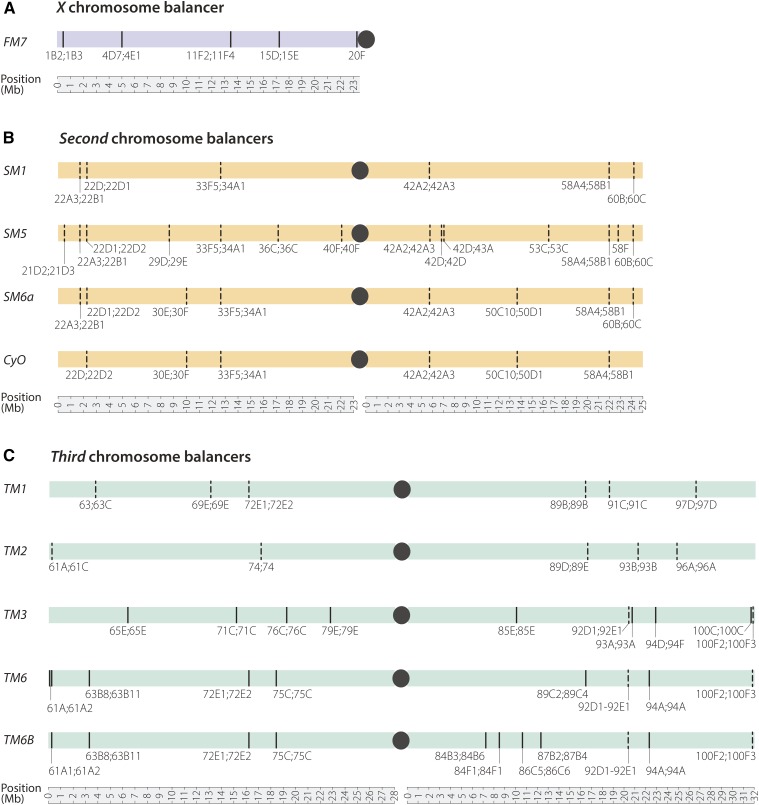
Inversion breakpoints for commonly used X, second, and third chromosome balancers. Breakpoints that have been molecularly identified are shown as solid lines; those that are estimates are shown as dashed lines; centromeres are represented by black dots; coordinates are based on release 6 of the *D. melanogaster* genome. (A) Inversion breakpoints of the X chromosome balancer *FM7* (Miller *et al.* 2016a). (B) Inversion breakpoints of four commonly used second chromosome balancers. (C) Inversion breakpoints of five commonly used third chromosome balancers, including the three balancers sequenced in this study.

An appreciation that single crossovers can occur distal to the 65D inversion on *TM3* also has practical purposes for long-term maintenance of deleterious alleles in stocks. At least 550 stocks at the Bloomington Stock Center have a mutation, transgene insertion, or chromosomal deletion distal to 65D that could be lost by recombination with the *TM3* present. Although this number assumes that recombination can occur anywhere from the tip of *3L* to the 65D breakpoint, our data suggests an ∼2-Mb buffer over which recombination may be suppressed, potentially reducing the number of vulnerable alleles. Yet the practical implication remains that genetic components thought to be present on all nonbalancer chromosomes in a population may be present in only a subset of individuals in the population, or may have been moved to the balancer chromosome itself. Therefore, it would be prudent for researchers to check for the presence of the desired genetic element distal to 65D in any *TM3* stock before undertaking experiments. Furthermore, poorly balanced regions exist at the ends of other popular balancers—including *CyO*, *In(2LR)Gla*, and *TM1*—and these balancers should generally be avoided in constructing stocks with distally located genetic components (Figure 4).

 We also recovered evidence of double crossing over between *TM3* and *TM6B* and their normal sequence homologs. Two of the stocks with DCO events, *TM3-560* and *TM3-1614*, are unique in that they appear to be examples of the *TM3* balancer before *Sb^1^* and *Ser^1^* (*TM3-1614*) or before *Sb^1^* (*TM3-560*) were added to *TM3* through double crossing over in a triple-balanced female (Tinderholt 1960). Recovery of DCO events on these balancer chromosomes was not surprising, as similar exchanges were recently shown to occur within the inverted *In(1)dl-49* segment of the *X* chromosome balancer *FM7c*. DCO events on *FM7c* always replaced the female sterile *singed^*X2*^* (*sn^X2^*) allele with a wild-type copy of the gene, resulting in *sn^+^* progeny with reproductive advantages (Miller *et al.* 2016a). Similarly, the DCO events recovered in the *TM3-504* and *TM6B-587* stocks created a small duplication and the elimination of three frameshifting deletions, respectively, each of which may confer selective advantages.

 The precise identification of inversion breakpoints, and the knowledge that rare DCO events are possible within inverted segments, should encourage researchers to carefully consider the proper balancer to use when keeping any allele over a balancer for a long period of time. We suggest using a balancer with an inversion breakpoint as close to the allele of interest as possible to prevent loss through double crossing over (Figure 4). In cases when this is not feasible, then keeping multiple copies of a stock along with periodic validation of the allele is likely in order.

*Drosophila* has a rich history. It has been over 100 yr since Morgan began to demonstrate the power of this tiny fly as a potent tool for scientific inquiry (Muller 1946; Sturtevant 2001). The success and rapid progress of experimentation in *Drosophila* today relies on genetic tools that have been built over the past century. Balancers have been especially important to the development of *Drosophila* as a genetic model organism. Molecular characterization of balancers helps explain how they work, how they vary, and what their inherent limitations are. This study endeavors to help *Drosophila* geneticists make better use of these invaluable tools.

## Supplementary Material

Supplemental material is available online at www.g3journal.org/lookup/suppl/doi:10.1534/g3.116.029330/-/DC1
