# Supplementary material for: Third Chromosome Balancer Inversions Disrupt Protein-Coding Genes and Influence Distal Recombination Events in Drosophila melanogaster
Source: G3 (Bethesda). 2016 Jun 28;6(7):1959–67. doi: 10.1534/g3.116.029330 (PMC4938649; doi:10.1534/g3.116.029330)
Supplement: Supplementary file 1 [file 1959TableS1.pdf]

Table S1. PCR primers used to validate selected inversion breakpoints

Breakpoint	Balancer	F Primer Seq	R Primer Seq	Annealing Temp
65D-85F	*TM3*	CGATGGACAGAAGCAACAGA	ATCACCAGGACTACGGCAAC	60
65D-85F	*TM3*	TAATTCCTGTGAGCGACGTG	TAATGGGCATCAAGCATACG	60
71B-94D	*TM3*	TGGACGAACAAGCTAAACGA	TCTAAAATGCCCATCCAACC	60
71B-94D	*TM3*	AACAGCTCTTGAGGCGAGAC	TACACGAGTTTTGGCAGACG	60
76B-92F	*TM3*	GTAAGGGTTCCCTGGATGGT	GGCGATCAAACAACCAAAGT	60
76B-92F	*TM3*	TCAGGTGATGTGCTGGAATC	AGGAAGATCCCGCAATAGGT	60
79F-100D	*TM3*	CCTCCGAAACGCATTGTATT	TGCAGTTGGATAGGTTCGTG	60
79F-100D	*TM3*	ATTTGGATCCATTCCGTTGA	AACAGGGCGGCTACTTGTTA	60
61B-87B	*TM6B*	TCACTTTAGCAGGTTCCATCG	TTGAACCCGAAATGGCTTTA	60
61B-87B	*TM6B*	TTGACAGGGTGGTCCAATTA	AATTTGCTTCGCAATGAAGG	60
75D-94A	*TM6, TM6B*	AAATTGCCGATCAAAAGGTG	ATTAATTGGCCCAGGACCTC	60
75D-94A	*TM6, TM6B*	AACCCACGAGTCCCCTAACT	ACCCCGAAGTGTGCAGTATC	60
61A-89C	*TM6*	GCACACTCCGCACACTTG	CGGGTAAGAGCATGACCAAT	63
61A-89C	*TM6*	TTTGAGCTGCACTCTTGCAC	TTCCCCATCAACTCCTCTCA	61
61A-89C	*TM6*	ACCCCATTTTTCACTTGCTG	ACGTGTGGGGTCACTAGAGG	61
