# Supplementary material for: Third Chromosome Balancer Inversions Disrupt Protein-Coding Genes and Influence Distal Recombination Events in Drosophila melanogaster
Source: G3 (Bethesda). 2016 Jun 28;6(7):1959–67. doi: 10.1534/g3.116.029330 (PMC4938649; doi:10.1534/g3.116.029330)
Supplement: Supplementary file 2 [file 1959TableS2.pdf]

Table S2. Stocks sequenced in this study

Bloomington ID	Current Genotype
**TM3 Stocks**	
N/A	*wg*$^{Sp-1}$*/SM6a, Pr*^{1} *Dr*^{1}*/TM3, Sb*^{1} *Ser*^{1}
120	*TM3, ry*^{RK} *Sb*^{1} *Ser*^{1}*/TM6B, Tb*^{1}
500	*eg*^{1}*/TM3, Sb*^{1} *Ser*^{1}
504	*amd*^{21} *Bl*^{1}*/CyO; DCTN1-p150*^{1}*/TM3, Sb*^{1} *Ser*^{1}
560	*Pri*^{1} *Dr*^{1}*/TM3*
1614	*y*^{1} *w***; TM3 y*$^{+}$ *Ser*^{1}*/ Sb*^{1}
1679	*Dp(1;Y)B*^{S}*; dsx*^{1} *p*^{p}*/TM3, Sb*^{1}
2053	*twr*^{1} *red*^{1} *e*^{1}*/TM3, Sb*^{1} *Ser*^{1}
2098	*sas*^{15} *p*^{p} *cu*^{1}*/TM3, Sb*^{1} *Ser*^{1}
2198	*Df(3R)ro80b, st*^{1} *e*^{1}*/TM3, Sb*^{1}
2485	*ru*^{1} *h*^{1} *Diap1*^{1} *st*^{1} *cu*^{1} *srp*^{3} *sr*^{1} *e*^{s} *ca*^{1}*/TM3, Sb*^{1} *Ser*^{1}
3251	*CyO, l(2)DTS513*^{1}*/l(2)****; BicF*^{1}*/TM3, Sb*^{1} *Ser*^{1}
5457	*hkb*^{2} *p*^{p}*/TM3, p*$^{+}$ *Sb*^{1}
8852	*mwh*^{2} *ru*^{1} *kni*$^{ri-1}$*/TM3, mwh*^{2} *ru*^{1} *Sb*^{1}
9013	*l(3)SG43*^{1} *red*^{1}*/TM3, Sb*^{1} *Ser*^{1}
22239	*y*^{1} *w***; P{y*$^{+t7.7}$*=Mae-UAS.6.11}Dpit47*^{LA00491}*/CyO; l(3)****/TM3, Sb*^{1} *Ser*^{1}
24759	*w***; sna*^{Sco}*/CyO; P{w*$^{+mC}$*=ninaD-GAL4.W}3/TM3, Sb*^{1}
38418	*w***; P{w*$^{+mW.hs}$*=GawB}nubbin-AC-62/CyO; P{w*$^{+mC}$*=UAS-Nslmb-vhhGFP4}3/TM3, Sb*^{1} *Ser*^{1}
**TM6 Stocks**	
N/A	*+/TM6, Ubx*
**TM6B Stocks**	
120	*TM3, ry*^{RK} *Sb*^{1} *Ser*^{1}*/TM6B, Tb*^{1}
587	*Sb*^{Spi}*/TM6B, Tb*^{1}
2188	*w*^{1118}*; Scr*^{4} *red*^{1} *e*^{1}*/TM6B, Tb*^{1}
